# An Optimization-Based Approach to Radar Image Reconstruction in Breast Microwave Sensing

**DOI:** 10.3390/s21248172

**Published:** 2021-12-07

**Authors:** Tyson Reimer, Stephen Pistorius

**Affiliations:** 1 Department of Physics and Astronomy, University of Manitoba, Winnipeg, MB R3T 2N2, Canada; stephen.pistorius@umanitoba.ca; 2 CancerCare Manitoba Research Institute, University of Manitoba, Winnipeg, MB R3E 0V9, Canada

**Keywords:** microwave imaging, breast microwave imaging, image reconstruction, radar imaging

## Abstract

Breast microwave sensing (BMS) has been studied as a potential technique for cancer detection due to the observed microwave properties of malignant and healthy breast tissues. This work presents a novel radar-based image reconstruction algorithm for use in BMS that reframes the radar image reconstruction process as an optimization problem. A gradient descent optimizer was used to create an optimization-based radar reconstruction (ORR) algorithm. Two hundred scans of MRI-derived breast phantoms were performed with a preclinical BMS system. These scans were reconstructed using the ORR, delay-and-sum (DAS), and delay-multiply-and-sum (DMAS) beamformers. The ORR was observed to improve both sensitivity and specificity compared to DAS and DMAS. The estimated sensitivity and specificity of the DAS beamformer were 19% and 44%, respectively, while for ORR, they were 27% and 56%, representing a relative increase of 42% and 27%. The DAS reconstructions also exhibited a hot-spot image artifact, where a localized region of high intensity that did not correspond to any physical phantom feature would be present in an image. This artifact appeared like a tumour response within the image and contributed to the lower specificity of the DAS beamformer. This artifact was not observed in the ORR reconstructions. This work demonstrates the potential of an optimization-based conceptualization of the radar image reconstruction problem in BMS. The ORR algorithm implemented in this work showed improved diagnostic performance and fewer image artifacts compared to the widely employed DAS algorithm.

## 1. Introduction

Improving the potential of microwave-based breast cancer detection has been an active area of investigation over the past two decades [1,2]. The observed contrast in the microwave properties of cancerous and healthy breast tissues [3,4,5,6] has sparked research into the use of breast microwave sensing (BMS) as a diagnostic tool to be used for breast cancer screening or as a complementary technique to mammography [1,2]. Several research groups have developed BMS systems and evaluated these systems using both phantom and patient data [7,8,9,10,11,12]. Despite the progress made in BMS research over the past two decades, several challenges remain before the technique can be considered for routine clinical use.

The two families of image reconstruction algorithms used in BMS today are radar-based and tomographic techniques. Microwave tomography typically aims to reconstruct a quantitative image of the dielectric properties of the breast, and does using the full electromagnetic scattering model [13,14,15,16]. While some assumptions may be made to simplify the inverse problem in tomography, using the full model is computationally expensive and presents challenges when applying these methods to experimental data [13,14,15,16].

Radar-based techniques have seen widespread use and have been used in several clinical trials [7,8,9,10,11,12]. These techniques assume a relatively straightforward radar signal model and are computationally inexpensive but typically neglect many aspects of the physics of microwave scattering. Radar-based reconstruction methods produce qualitative images of the breast, in which regions of high reflectivity are displayed. These techniques include the delay-and-sum beamformer, which has been used in several clinical studies [17]. Several other reconstruction methods have been derived from the delay-and-sum beamformer, including the delay-multiply-and-sum beamformer [18], which has been demonstrated to reduce both background and high-intensity clutter to accurately localize the tumour response in patient data [19].

Current microwave tomography and radar-based reconstruction methods both face challenges. This work presents a novel radar-based reconstruction method that conceptualizes the radar reconstruction approach as an optimization problem. The novel reconstruction method was used to produce images of an array of 3D-printed MRI-derived breast phantoms using measurements obtained with a preclinical BMS system. A dataset consisting of 200 scans of the phantoms was produced and is the third generation in the University of Manitoba Breast Microwave Imaging Dataset (UM-BMID, the original dataset was first presented in [20]) and was made open access. This dataset was used to compare the images produced with the novel method to those produced using the standard delay-and-sum and delay-multiply-and-sum beamformers. The signal-to-clutter ratio and localization error of the reconstructions were compared, and the diagnostic sensitivity and specificity were evaluated using the dataset. This optimization conceptualization allows for further implementation of improved physics modelling within the reconstruction algorithm. This article discusses the observed improvements in image quality and diagnostic performance using the novel optimization-based reconstruction method and the potential for future improvements through additional physics modelling.

The novelty of this work is three-fold: (1) an image reconstruction algorithm based on a reframing of the radar image reconstruction problem within an optimization framework, (2) an open-access dataset with well-understood positioning uncertainties, and extensive reference scan and data collection documentation for the evaluation of image reconstruction methods, specifically designed to isolate the effect of tumour size on tumour detection, and (3) estimates of the specificity of the literature’s standard DAS and DMAS methods (in addition to the novel reconstruction algorithm presented herein) using a relatively large dataset of 200 phantom scans and the identification of an image artifact present in reconstructions produced by the DAS and DMAS beamformers.

## 2. Materials and Methods

### 2.1. Standard Radar-Based Image Reconstruction Methods

The delay-and-sum (DAS) beamformer [17] is the standard method for radar-based image reconstruction in breast microwave sensing and has been used as the basis for the development of several derivative methods, including the delay-multiply-and-sum (DMAS) [18], improved delay-and-sum [21], coherence factor delay-and-sum [22], channel-ranked delay-and-sum [23], and the iterative delay-and-sum [24]. DAS reconstructs an image of the breast through summation of the individual radar signals acquired during the scan protocol. In a monostatic system, this can be expressed as

(1) $$I\left(r\right)=\sum_{a}\sum_{f}S\left(a,f\right)e^{-2\pi jf2t(r,a)}$$

where S(a,f) is the measured S${}_{11}$ signal at antenna position a and frequency *f*, I(r) is the image intensity at position r, and t(r,a) is the one-way time-delay for the signal to propagate from the antenna’s position a to the position r. Assuming a homogeneous propagation speed along the propagation path, the time-delay is modelled as

(2) $$t\left(r,a\right)=\frac{\left|r-a\right|}{v}$$

where *v* is the (homogeneous) propagation speed.

The DMAS beamformer reconstructs an image through signal-pair multiplication followed by summation,

(3) $$I\left(r\right)=\sum_{a}\sum_{a^{\prime }}\sum_{f}S\left(a,f\right)e^{-2\pi jf2t(r,a)}S\left(a^{\prime },f\right)e^{-2\pi jf2t(r,a^{\prime })}.$$

This algorithm, originally proposed for application in radar-based breast microwave imaging, has also been applied to ultrasound imaging [25]. The beamformer computes the autocorrelation function at each antenna measurement position, a, but excludes the autoproduct terms [25].

Both phantom [26] and patient [19] studies have demonstrated that the DMAS beamformer outperforms the delay-and-sum beamformer and other methods, including the improved delay-and-sum, coherence factor delay-and-sum, and channel-ranked delay-and-sum beamformers, with respect to clutter reduction and tumour localization accuracy [19,26].

### 2.2. The Optimization-Based Radar Image Reconstruction Algorithm

Existing radar-based image reconstruction techniques perform one-step image reconstruction, as in DAS and DMAS, where the image is produced in one mathematical step. While these techniques allow for rapid reconstruction, they do not allow nonlinear components to be incorporated into the signal model. The implicit forward signal model assumed in the DAS beamformer is given by

(4) $$S^{\mathrm{fwd}}\left(a,f,\sigma \right)=\int dr\sigma \left(r\right)e^{-2\pi jf2t(r,a)}$$

where $S^{\mathrm{fwd}}\left(a,f,\sigma \right)$ is the forward model S-parameter obtained at measurement position a, frequency *f*, due to reflectivity profile σ. The DAS beamformer aims to reconstruct this reflectivity profile, σ(r), as the image I(r).

This model assumes plane-wave propagation from the antenna to each scattering point and from each scattering point to the antenna. The model assumes no signal losses due to distance-based attenuation, partial transmission at interfaces, and losses due to lossy propagation media. The model assumes an isotropic, point-source antenna with a uniform gain as a function of frequency and also assumes a uniform propagation speed throughout the imaging domain. These assumptions are all violated in a BMS system, and as a result, the performance of the DAS beamformer and its derivatives are constrained.

However, this radar forward model can be also used to produce an optimization problem,

(5) $$\sigma_{\mathrm{img}}=\arg\min_{\sigma }\left(\sum_{a}\sum_{f}|S^{\mathrm{fwd}}\left(a,f,\sigma \right)-S^{\mathrm{expt}}\left(a,f\right){|}^{2}\right)$$

where $\sigma_{\mathrm{img}}$ is the reflectivity profile displayed in the reconstructed image and $S^{\mathrm{expt}}\left(a,f\right)$ is the experimentally measured S-parameter at measurement position a and frequency *f*, and where the sum-of-squares,

(6) $$l\left(\sigma \right)=\sum_{a}\sum_{f}|S^{\mathrm{fwd}}\left(a,f,\sigma \right)-S^{\mathrm{expt}}\left(a,f\right){|}^{2}$$

was used as the loss function, l(σ).

To solve this optimization problem, we employed the gradient descent optimizer to find $\sigma_{\mathrm{img}}$,

(7) $$\sigma^{\left(n+1\right)}=\sigma^{\left(n\right)}-\alpha \bar{\nabla_{\sigma }l\left(\sigma \right)}$$

where the conjugate of the gradient (with respect to the reflectivity profile, $\nabla_{\sigma }$) of the loss function is used due to $S^{\mathrm{fwd}},S^{\mathrm{expt}}\in C$ [27].

Let us explore the relationship between the DAS reconstruction algorithm and this optimization problem formulation. Assume that the measured S${}_{11}$ is at only one position, $a_{0}$, and one frequency $f_{0}$, to examine the exact solution for σ(r), assuming the implicit forward model in Equation (4), by writing:

(8) $$S^{\mathrm{expt}}\left(f_{0},a_{0}\right)=S^{\mathrm{fwd}}\left(f_{0},a_{0},\sigma \right)$$

where $S^{\mathrm{fwd}}\left(f_{0},a_{0},\sigma \left(r\right)\right)$ is given as in Equation (4), so that

(9) $$S^{\mathrm{expt}}\left(f_{0},a_{0}\right)=\int_{V}dr\sigma \left(r\right)e^{-2\pi jf_{0}2t\left(r,a_{0}\right)}.$$

The reflectivity profile that solves this equation, $\sigma^{D}\left(r\right)$, is

(10) $$\sigma^{D}\left(r\right)=\left(\frac{1}{V}\right)S^{\mathrm{expt}}\left(f_{0},a_{0}\right)e^{2\pi jf_{0}2t\left(r,a_{0}\right)}$$

where *V* is the volume of the integration domain.

To prove this, substitute Equation (10) into Equation (9), to obtain

(11) $$S^{\mathrm{expt}}\left(f_{0},a_{0}\right)\overset{?}{=}S^{\mathrm{fwd}}\left(f_{0},a_{0},\sigma^{D}\right)$$

(12) $$\Rightarrow S^{\mathrm{expt}}\left(f_{0},a_{0}\right)\overset{?}{=}\int_{V}dr\left[\left(\frac{1}{V}\right)S^{\mathrm{expt}}\left(f_{0},a_{0}\right)e^{2\pi jf2t(r,a_{0})}\right]e^{-2\pi jf_{0}t\left(r,a_{0}\right)}$$

(13) $$\Rightarrow S^{\mathrm{expt}}\left(f_{0},a_{0}\right)\overset{?}{=}\left(\frac{1}{V}\right)S^{\mathrm{expt}}\left(f_{0},a_{0}\right)\int_{V}dr$$

(14) $$\Rightarrow S^{\mathrm{expt}}\left(f_{0},a_{0}\right)\overset{?}{=}\left(\frac{1}{V}\right)S^{\mathrm{expt}}\left(f_{0},a_{0}\right)V$$

and the factor of *V* in the numerator of the right-hand side cancels with that in the denominator, and so the left-hand side and right-hand sides are identical, demonstrating that Equation (10) is a solution to the cost function l(σ) assuming the forward model to be implicit to DAS when one measurement position $a_{0}$ and one frequency $f_{0}$ are used,

(15) $$\Rightarrow S^{\mathrm{expt}}\left(f_{0},a_{0}\right)=S^{\mathrm{fwd}}\left(f_{0},a_{0},\sigma^{D}\left(r\right)\right).$$

The solution $\sigma^{D}\left(r\right)$ in Equation (10) is also that given by the DAS beamformer, within a normalization factor of 1/V. For a single-position, single-frequency measurement, DAS therefore completely reconstructs the reflectivity profile σ(r) so as to minimize the loss function l(σ) (in this case, l(σ)=0). However, no current BMS system uses only a single-position, single-frequency measurement to reconstruct an image (due primarily to the non-unique time delays—i.e., a given time delay may correspond to many regions in the spatial domain). If we consider a BMS system that uses a single-position, dual-frequency measurement ($f_{0}$ and $f_{1}$), we observe that the reflectivity profile produced by the DAS beamformer, $\sigma^{D}\left(r\right)$ as described in Equation (10), no longer reproduces the experimental measured S-parameter, $S^{\mathrm{expt}}$, for either frequency. To illustrate this, consider the reflectivity profile produced by DAS using the information from the dual-frequency measurement,

(16) $$\sigma^{D}\left(r\right)=\left(\frac{1}{V}\right)\left[S^{\mathrm{expt}}\left(f_{0},a_{0}\right)e^{2\pi jf_{0}2t\left(r,a_{0}\right)}+S^{\mathrm{expt}}\left(f_{1},a_{0}\right)e^{2\pi jf_{1}2t\left(r,a_{0}\right)}\right].$$

If this relationship is used in Equation (9), we obtain,

(17) $$\begin{array}{c} S^{\mathrm{expt}}\left(f_{0},a_{0}\right)=\left(\frac{1}{V}\right)\int_{V}dr\left[S^{\mathrm{expt}}\left(f_{0},a_{0}\right)e^{2\pi jf_{0}2t\left(r,a_{0}\right)}+S^{\mathrm{expt}}\left(f_{1},a_{0}\right)e^{2\pi jf_{1}2t\left(r,a_{0}\right)}\right]\cdot \\ \cdot e^{-2\pi jf_{0}2t\left(r,a_{0}\right)}, \end{array}$$

for $S^{\mathrm{expt}}\left(f_{0},a_{0}\right)$. Neglecting the prefactor 1/V and simplifying the integrand, this becomes

(18) $$S^{\mathrm{expt}}\left(f_{0},a_{0}\right)=S^{\mathrm{expt}}\left(f_{0},a_{0}\right)+\int_{V}dr\;S^{\mathrm{expt}}\left(f_{1},a_{0}\right)e^{2\pi jf_{1}2t\left(r,a_{0}\right)}e^{-2\pi jf_{0}2t\left(r,a_{0}\right)}$$

(19) $$\Rightarrow S^{\mathrm{expt}}\left(f_{0},a_{0}\right)\neq S^{\mathrm{fwd}}\left(f_{0},a_{0},\sigma^{D}\left(r\right)\right).$$

This demonstrates that the experimental S-parameters are not perfectly reconstructed when the DAS beamformer is used with multiple measurements. Therefore, the gradient descent algorithm may be able to provide an improvement with respect to the loss function l(σ). This mathematical discussion motivated the development of the optimization-based reconstruction approach, as the ORR may improve upon the DAS reconstruction, with respect to the loss function l(σ).

The initial reflectivity profile estimate for all reconstructions produced in this work was a uniform map of zeroes, $\forall r,\sigma^{\left(0\right)}\left(r\right)=0$. The reconstruction procedure was stopped when the relative change in the loss function was less than 0.1%, i.e.,

(20) $$\frac{l\left(\sigma^{\left(n+1\right)}\right)-l\left(\sigma^{\left(n\right)}\right)}{l(\sigma^{\left(n\right)})}\times 100\%\leq 0.1\%.$$

### 2.3. Experimental Dataset: UM-BMID Gen-3

The open-access dataset developed by our group, the University of Manitoba Breast Microwave Imaging Dataset (UM-BMID) [20], has been used in past work for machine learning-based tumour detection [28,29]. The dataset presented in [20] consisted of two generations, and while the data are well-suited for machine learning investigations, the challenges in target positioning uncertainty and reference scan acquisition limit the utility of the first- and second-generation datasets for image-based analysis.

The third generation of UM-BMID was developed to complement the existing dataset generations and facilitate image-based tumour detection analysis in experimental BMS. This dataset was generated using scans of 3D-printed phantoms (first described in [30] and [20]) performed with the BMS system described in [31]. One modification to the system in [31] was made in the completion of this dataset. The vector network analyzer (VNA) in [31] was a Planar 804/1 (Planar 804/1, Copper Mountain Technologies, Indianapolis, IN, USA), and operated over 1–8 GHz, while the VNA used to produce this dataset was the C1209 model (C1209, Copper Mountain Technologies, IN, USA) and operated over 1–9 GHz. The C1209 was used due to its improved dynamic range, an improved output power of 15 dBm (compared to 10 dBm for the Planar 804/1) and larger frequency bandwidth than the Planar 804/1. The scan protocol was otherwise the same—measurements were performed at 1001 frequencies over the 1–9 GHz bandwidth, and the antenna was rotated to 72 positions equally spaced along a circular trajectory spanning 355${}^{\circ }$.

**Figure 1:**
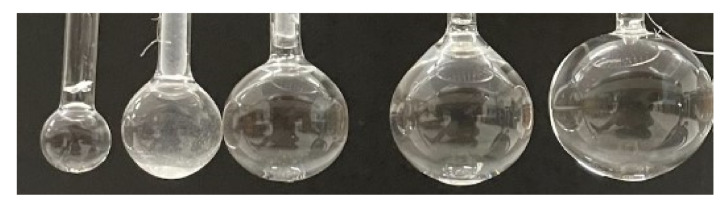
The glass tumour bulbs (filled with saline solution) used as tumour surrogates in the breast phantoms.

The third generation dataset consisted of scans of 20 unique healthy phantom tissue geometries. Five tumour-containing scans were performed for each unique phantom. These five scans differed only with respect to the size of the tumour—the tumours were placed at the same position in each of these five scans. Tumours of 10, 15, 20, 25, and 30 mm diameters were used, as displayed in Figure 1.

Each tumour-containing scan had two unique reference scans and one nonunique reference scan. The unique references were of the same phantom tissue geometry, excluding the tumour (referred to as the healthy reference), performed immediately after the target tumour-containing scan, and of the same phantom tissue geometry, excluding the tumour and the fibroglandular components (referred to as the adipose reference), performed immediately after the healthy reference. The nonunique reference was an empty chamber scan. The healthy reference scans were performed to facilitate the visualization of the effects of the fibroglandular inclusion, and the adipose reference scans were performed to facilitate image reconstruction under conditions of ideal surface reflection subtraction. The adipose reference scan subtraction allows for manual removal of the reflections due to the air–tissue interface, serving as an experimental (but not clinically practical) method for ideal surface reflection subtraction. While skin suppression is an active area of research [2], this allows the relatively large reflections due to the air–tissue interface to be suppressed to visualize the interior tissue components.

Table 1 compares the novel third-generation dataset to the previously presented second-generation dataset. The third generation reduced target positioning uncertainty by nearly a factor of three and included more reference scans to facilitate more informative image analyses.

This dataset consists of 120 unique phantom scans and is publicly available at https://bit.ly/UM-bmid, accessed on 2 December 2021. All Python code used to perform the analysis presented in this work and to create the figures displayed in this article is openly accessible https://github.com/TysonReimer/ORR-Algorithm, accessed on 2 December 2021.

### 2.4. Image Quality Analysis and Diagnostic Performance Estimation

The signal-to-clutter ratio (SCR) and localization error (LE) were calculated for each reconstruction. These image quality metrics were defined as in [24] and measure image contrast (SCR) and image accuracy (LE). The SCR (in dB) was defined as

(21) $$\mathrm{SCR}=20\log_{10}\left(\frac{T_{\max}}{C_{\max}}\right)$$

where $T_{\max}$ is the maximum response in the target (tumour) region and $C_{\max}$ is the maximum response in the clutter (nontumour) region.

The LE was defined as

(22) $$\mathrm{LE}=|r_{\max}-r_{\mathrm{tum}}|$$

where $r_{\max}$ is the location of the maximum intensity response within the image and $r_{\mathrm{tum}}$ is the known location of the center of the tumour.

These metrics were determined for each reconstruction produced by the DAS, DMAS, and ORR algorithms.

An automated and quantitative tumour-detection metric was used in this work to evaluate the diagnostic sensitivity and specificity of the DAS, DMAS, and ORR methods. The tumour-detection criteria used in this work were the same as described in [32]. If the SCR of an image was greater than 1.5 dB, then that image was labelled as containing a tumour because a large SCR indicates a high-contrast, localized region within the image. For reconstructed images of healthy phantoms, this was the only criterion used to evaluate the label of the image—all healthy images with SCR≥1.5 dB were labelled as false positives, and all healthy images with SCR<1.5 dB were labelled as true negatives. For reconstructed images of tumour-containing phantoms, if the SCR<1.5 dB, the image was labelled as a false negative. If the SCR≥1.5 dB, the localization error was also evaluated. If the localization error was $\mathrm{LE}\leq r_{\mathrm{tum}}+\delta_{r}$, where $r_{\mathrm{tum}}$ was the known radius of the tumour and $\delta_{\rho }$ was the uncertainty in the polar position of the tumour, then the image was labelled as a true positive, because the highest-intensity point in the image was found to be within the expected tumour region. If $\mathrm{LE}>r_{\mathrm{tum}}+\delta_{r}$, then the image was also labelled as an incorrect prediction and was treated as a false negative when calculating the sensitivity of the reconstruction method.

## 3. Results

**Figure 2:**
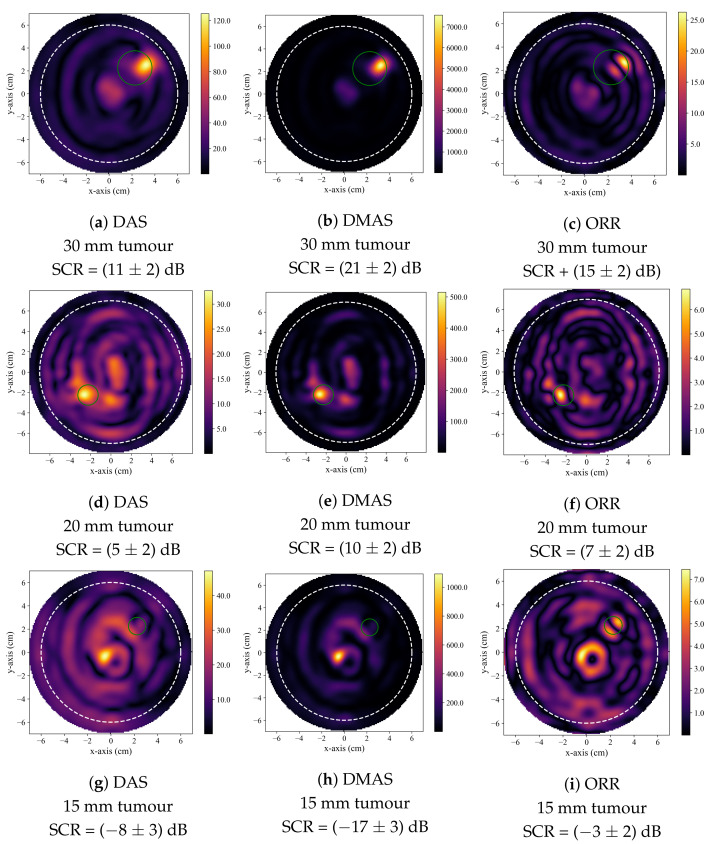
Typical reconstructed images produced by DAS in (**a**,**d**,**g**), by DMAS in (**b**,**e**,**h**), and by the ORR in (**c**,**f**,**i**). The first row displays reconstructions of a phantom containing a 30 mm tumour, the second of a phantom containing a 20 mm tumour, and the third of a phantom containing a 15 mm tumour. In all reconstructions, the dashed white circle indicates the approximate outer boundary of the breast phantom, and the solid green circle indicates the known tumour position.

Figure 2 displays typical reconstructions produced by the DAS, DMAS, and ORR algorithms. Figure 2a–c displays reconstructions of a 30 mm diameter tumour and is typical of a case where all three beamformers successfully identified the presence of the tumour inclusion based on the quantitative criteria described in Section 2.4. Figure 2d–f displays reconstructions of a 20 mm tumour and demonstrates the generally observed trend, where the DMAS and ORR reconstructions had higher SCR than the DAS reconstructions. Figure 2g–i displays reconstructions of a 15 mm tumour and demonstrates typical reconstructions produced by the beamformers when the tumour is not accurately detected. While the SCR of the DAS and DMAS reconstructions are negative, the true tumour location does not correspond to a visual image feature, and the central high-intensity localized region of the reconstructions visually appears similar to that of a tumour response (as in Figure 2d–f). This *hot-spot* artifact was present in many DAS and DMAS reconstructions and obfuscated the true tumour response. Notably, the ORR reconstruction does not have a single localized high-intensity region, as in the case of the DAS and DMAS reconstructions, but instead has two distinct relatively high-intensity regions—one occurring near the center of the image (as in the DAS and DMAS reconstructions) and the other at the known location of the tumour.

**Figure 3:**
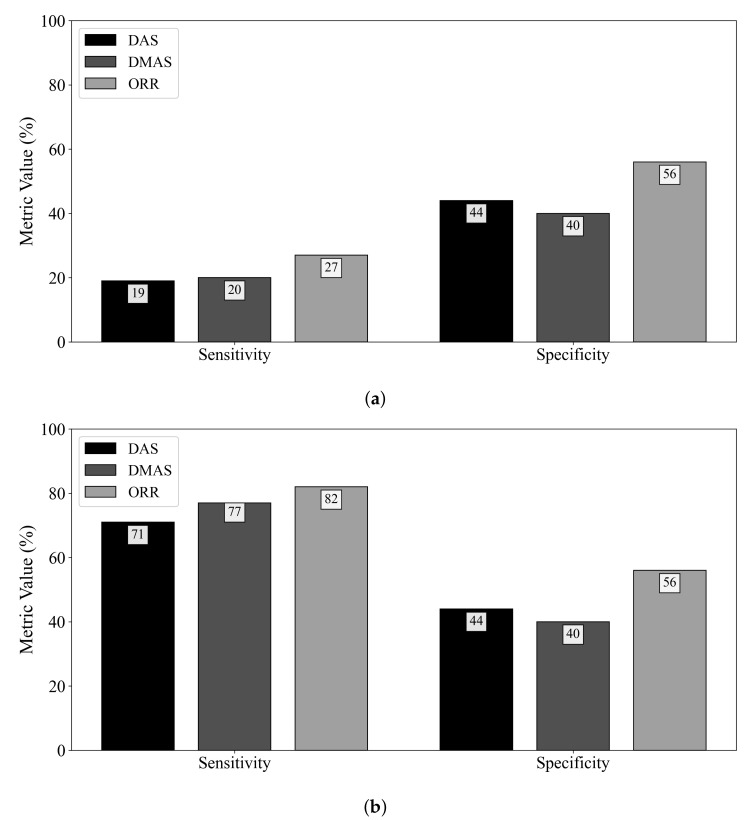
Sensitivity and specificity of the DAS, DMAS, and ORR reconstruction methods evaluated on UM-BMID Gen-3 when (**a**) the adipose-only scan is used as the reference and (**b**) when the adipose-fibroglandular scan is used as the reference.

Figure 3 displays the diagnostic sensitivity and specificity obtained by the DAS, DMAS, and ORR algorithms when (a) an adipose reference scan is used for ideal skin suppression and (b) when an adipose and fibroglandular reference scan is used for ideal skin and clutter suppression. The subtraction of a reference scan containing both adipose and fibroglandular tissues allows for the removal of all responses not due to the tumour (neglecting secondary and higher-order scattering). While this suppression is not clinically practical, it allows for insight into the effects of the fibroglandular inclusions on the diagnostic performance of the reconstruction methods. When ideal skin suppression is used, as displayed in Figure 3a, no beamformer has a practical sensitivity. If adipose and fibroglandular reference subtraction is performed, the beamformers have improved performance that is within the range of reported sensitivity estimates in the literature (74–100% in [8,9,32,33,34,35,36,37]). The specificity of the DAS and DMAS beamformers was observed to be worse than a random classifier acting on images of healthy breasts. Only the ORR algorithm achieved a specificity better than 50%.

**Figure 4:**
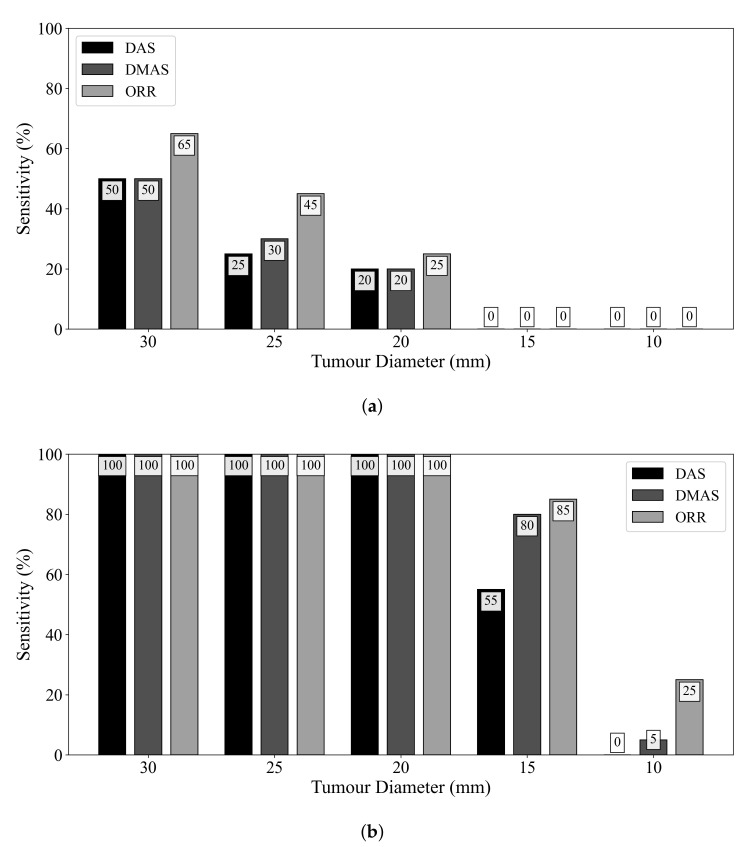
Sensitivity of the DAS, DMAS, and ORR reconstruction methods as a function of tumour size, evaluated on UM-BMID Gen-3 when (**a**) the adipose-only scan is used as the reference and (**b**) when the adipose-fibroglandular scan is used as the reference.

Figure 4 displays the influence of tumour size on diagnostic sensitivity for the three beamformers. The ORR algorithm had a 50% relative increase in sensitivity compared to the DMAS beamformer for detecting tumours of 25 mm diameter. Even when ideal skin suppression was used, all reconstruction methods failed to detect any 15 or 10 mm diameter lesions. When an adipose-fibroglandular scan was used as the reference for subtraction, all beamformers detected all lesions of at least 20 mm diameter. In all cases, the ORR outperformed both the DAS and DMAS beamformers.

## 4. Discussion

### 4.1. Image Artifacts in DAS, DMAS, and the Gradient Descent Method

**Figure 5:**
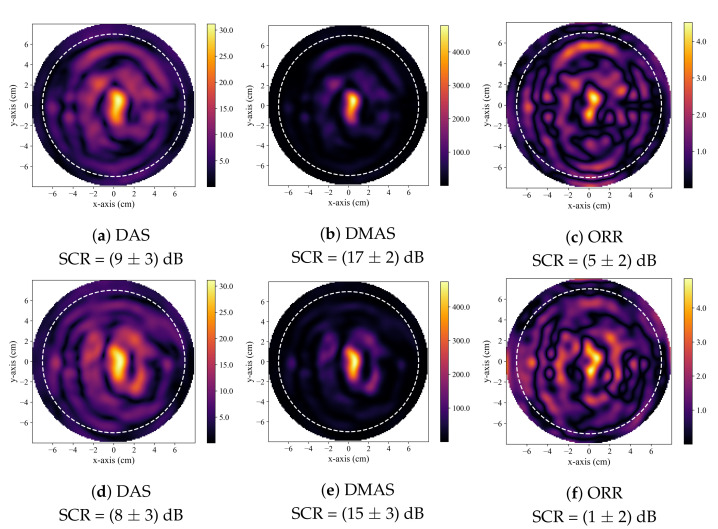
Typical reconstructed images produced by DAS in (**a**,**d**), by DMAS in (**b**,**e**), and by the ORR in (**c**,**f**). All reconstructions are of healthy, tumour-free phantoms. In all reconstructions, the dashed white circle indicates the approximate outer boundary of the breast phantom, and the solid green circle indicates the known tumour position.

A *hot-spot* artifact was observed in the reconstructions produced by both the DAS and DMAS beamformers. This artifact was observed as a localized high-intensity region within a reconstructed image that did not correspond to any physical phantom feature. The appearance of this artifact was similar to the appearance of a tumour response within an image. While this artifact can be observed in other published work that used the DAS and DMAS, to the best of the authors knowledge, this article is the first to identify and discuss its presence. Notably, the ORR reconstructions were not observed to have this artifact. Two typical cases of this artifact are displayed in Figure 5. The effect of the presence of this artifact in the DAS and DMAS reconstructions can be directly observed in the diagnostic specificity estimates.

### 4.2. The Diagnostic Performance of DAS, DMAS, and ORR

Only two studies in the literature have estimated the specificity of BMS techniques. Both studies used radar-based image reconstruction methods. One study [37] estimated the specificity to be 62% in a trial with 103 breast images from a patient trial. The other study estimated the specificity to be 20% and estimated the specificity using five phantoms [32]. Neither estimates were promising, and while the sensitivity of BMS has been estimated in several publications and has been reported to be between 74% and 100% [8,9,32,33,34,35,36,37], insufficient attention has been awarded to the specificity of the modality, which must be considered in tandem with the sensitivity.

The specificity results in this work demonstrate a qualitative observation—the DMAS beamformer tends to produce reconstructions with hot spots, even in the absence of a tumour. DMAS had the worst specificity (40%), while the ORR algorithm provided more than a 50% relative increase in specificity. DAS had a similarly poor specificity of 44%. Both DAS and DMAS were worse than a random classifier when predicting the presence of a tumour in healthy breasts. The hot-spot artifact discussed in Section 4.2 must be considered when evaluating image quality. While image artifacts may be difficult to quantify, the effects of this artifact on the specificity of the beamformers are clear from Figure 3.

**Figure 6:**
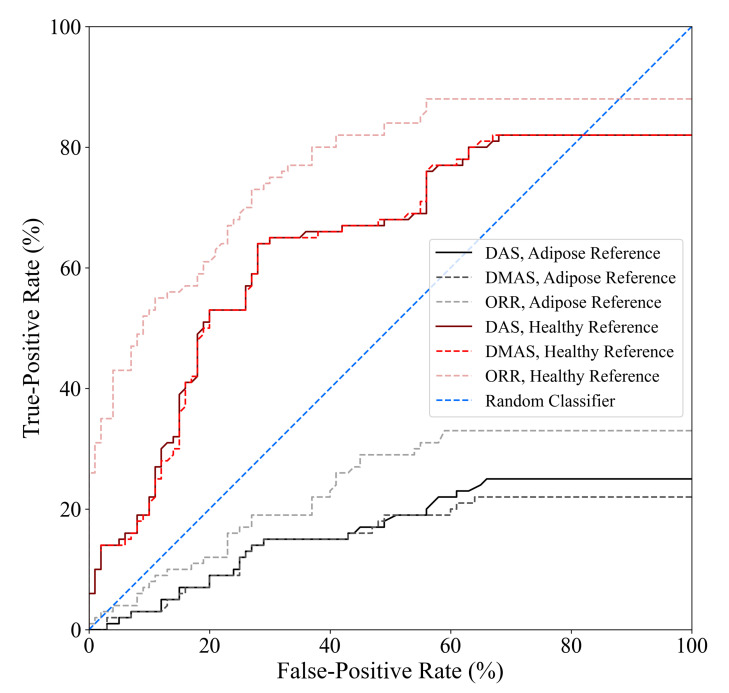
ROC curves of the DAS, DMAS, and ORR algorithms when either adipose-only phantom scans or healthy (adipose and fibroglandular) phantom scans are used as references for artifact suppression, determined using one thousand linearly spaced SCR thresholds between 0 and 30 dB. The ORR outperformed the DAS and DMAS beamformers for all SCR thresholds.

The tumour detection criteria used in this work, as defined in [32], requires two parameters to be defined *a priori*: (1) the SCR threshold used to determine whether an image is labelled as containing a tumour response and (2) the localization error threshold used to determine whether a high-intensity response labelled as a tumour response (due to the SCR threshold) belongs to the true tumour within the image. The localization error threshold used in this work was based on the known uncertainty in the positioning of the tumour surrogate within the imaging chamber, and the SCR threshold was the same as in [32], SCR ≥1.5 dB. The diagnostic performance estimates obtained in this analysis are sensitive to the selection of the SCR threshold. Figure 6 displays the receiver operating characteristic (ROC) curves of the DAS, DMAS, and ORR beamformers as a function of the SCR threshold used in the tumour detection criteria. One thousand linearly spaced SCR thresholds between 0 and 30 dB were used to compute each point on the curves. The ORR beamformer outperformed the DAS and DMAS reconstruction methods in all cases.

While the specificity of ORR is relatively poor compared to existing clinical modalities, it is similar to the 62% specificity reported in [37], and the relative increase in specificity compared to the literature standard DAS and DMAS beamformers is promising. No existing technique has been demonstrated to have specificity that would be clinically acceptable, but the relative improvement of 40% (compared to DMAS) demonstrates the potential of the ORR algorithm for BMS. The incorporation of improved physics modelling into the ORR method may further improve the specificity of the modality.

### 4.3. Advantages of Enhanced Physics Modelling in Radar-Based Image Reconstruction

This work demonstrates the advantages of the optimization conceptualization of the radar-based image reconstruction problem mathematically in Section 2.2 and empirically in Section 3. The optimization approach also allows for the incorporation of enhanced physics modelling to further improve the signal model used in the approach. The base radar forward model, also implicitly assumed by the DAS beamformer, in Equation (4), was used in the ORR presented in this work. This base model still reduced image artifacts and improved sensitivity and specificity compared to the DAS and DMAS beamformers.

Future work will explore the incorporation of improved physics modelling. The antenna beam pattern and frequency-dependent gain, system output signal, signal attenuation, non-uniform propagation speed, and multiple scattering can be incorporated into the radar signal model. Work performed with a maximum likelihood expectation maximization algorithm [38] demonstrated that modelling the antenna beam pattern improved reconstructions, and future work with the ORR will explore this further. Notably, this optimization approach allows for the incorporation of nonlinear enhanced physics. Multiple scattering effects are ignored in current radar-based reconstruction approaches, but second-order scattering could be included in the ORR through a modification in the signal model to

(23) $$\begin{array}{c} S^{\mathrm{fwd}}\left(a,f,\sigma \right)=\int dr\sigma \left(r\right)e^{-2\pi jf2t(r,a)}\\ +\int dr\sigma \left(r\right)e^{-2\pi jft(r,a)}\int dr^{\prime }\sigma \left(r^{\prime }\right)e^{-2\pi jf[t\left(r^{\prime },a\right)+t\left(r,r^{\prime }\right)]} \end{array}$$

where the second term models the second-order scattering.

This optimization-based reconstruction method provides the first opportunity in radar-based BMS image reconstruction to incorporate the multiple scattering effects into the reconstruction algorithm, and future work will examine the impact of this modelling on image quality and diagnostic performance.

## 5. Conclusions

This work describes a novel framework for image reconstruction in radar-based breast microwave imaging based on a complex optimization approach and presents results obtained using an implementation of this framework with the gradient descent optimizer and base radar signal model. The optimization-based radar reconstruction (ORR) algorithm was compared to the literature standard delay-and-sum (DAS) and delay-multiply-and-sum (DMAS) beamformers. A dataset of 200 scans of 3D-printed MRI-derived breast phantoms was created to compare the reconstructions produced by the DAS, DMAS, and ORR methods. The diagnostic performance of the ORR algorithm was better than the performance of DAS and DMAS with respect to sensitivity and specificity. The ORR algorithm had a relative increase in sensitivity of 42% and 35% compared to DAS and DMAS, respectively, and a relative increase in specificity of 27% and 40%, compared to DAS and DMAS. This work is the first to identify and describe the *hot-spot* image artifact present in DAS and (particularly) in DMAS reconstructions. This artifact appears as a localized high-intensity region within the image that resembles a tumour response but that does not belong to a physical phantom feature. This artifact was observed in reconstructions produced by both the DAS and DMAS beamformers, and the presence of this tumour-like artifact in the reconstructions of healthy phantoms resulted in poor specificity estimates for both beamformers. The ORR algorithm was not observed to produce reconstructions with this artifact. This work demonstrates the potential utility of the ORR algorithm as a novel methodology for image reconstruction in radar-based breast microwave sensing.

## Figures and Tables

**Table 1:** UM-BMID Gen-3 and Gen-2 comparison.

	Gen-3	Gen-2
Number of Unique Scans	120	1008
Number of Scans	200	1008
Adipose Reference Scans	Yes	No
Adipose-Fibroglandular Reference Scans	Yes	No
Scan Frequencies	1–9 GHz	1–8 GHz
Tumour Diameters (mm)	10, 15, 20, 25, 30	10, 20, 30
Positioning Uncertainty	4 mm	10 mm

## Data Availability

The data used in this study (UM-BMID Gen-3) can be accessed at https://bit.ly/UM-bmid, accessed on 2 December 2021. All Python code used to perform the analysis and produce the figures in this article can be accessed at https://github.com/TysonReimer/ORR-Algorithm, accessed on 2 December 2021.

## Abbreviations

The following abbreviations are used in this manuscript:

BMS	Breast microwave sensing
DAS	Delay-and-sum
DMAS	Delay-multiply-and-sum
ORR	Optimization-based radar reconstruction
VNA	Vector network analyzer
